# Intravenous Leiomyomatosis of the Uterus Extending to the Right Atrium: A Case Report

**DOI:** 10.3400/avd.cr.24-00084

**Published:** 2025-01-21

**Authors:** Kaori Katsumata, Yasunori Iida, Kento Kuroo, Yu Inaba, Takahisa Miki, Takashi Hachiya, Hideyuki Shimizu

**Affiliations:** 1 Department of Cardiovascular Surgery, Saiseikai Yokohamashi Tobu Hospital, Yokohama, Kanagawa, Japan; 2 Department of Cardiovascular Surgery, Keio University School of Medicine, Tokyo, Japan

**Keywords:** intravenous leiomyomatosis, syncope, cardiac tumor

## Abstract

Intravenous leiomyomatosis (IVL) remains scarcely reported, and complete tumor resection is the recommended treatment. Herein, we present a comprehensive review of the case of a 52-year-old woman who suffered from recurrent syncope episodes due to IVL with intracardiac extension to the right atrium. Partial tumor resection and postoperative hormone therapy were conducted first. However, the 6-month postoperative follow-up computed tomography scan revealed a tendency for the IVL to increase in size, and complete resection was conducted. In this article, we would like to emphasize that partial resection followed by hormone therapy is insufficient for IVL, and complete resection should be chosen.

## Introduction

Intravenous leiomyomatosis (IVL) is a benign tumor associated with uterine leiomyomas, but it occasionally acts aggressively with rapid growth and causes symptoms such as syncope. IVL with intracardiac extension is known as intracardiac leiomyomatosis (ICLM). Reports of ICLM are relatively rare. IVL occurs in 0.25%^1^^)^ of uterine leiomyomas, and ICLM occurs in 10%–40% of IVL.^2^^)^

Because of its rarity, there have long been various opinions on the treatment strategy for ICLM. Recently, however, there has been a growing consensus that complete resection should be aimed for, whether in a single- or 2-stage approach. On the other hand, there are some reports of cases where only the intracardiac tumor was partially resected and hormone therapy was administered for the residual tumor, resulting in no further growth.

Here, we present a case of ICLM in which we finally conducted total resection.

## Case Report

**Figure 1:**
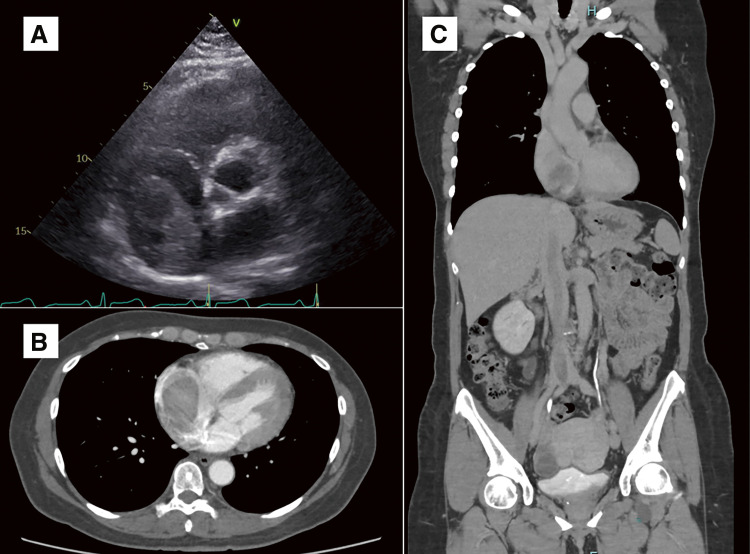
Fig. 1 (**A**) Preoperative TTE revealed a 6 × 4 cm moving structure within the right atrium. (**B**, **C**) Preoperative contrast-enhanced CT showed a 37-cm-long tumor extending from the right side of the uterine body through the right internal iliac vein and IVC to the right ventricle. TTE: Transthoracic echocardiography; IVC: inferior vena cava

A 52-year-old healthy and premenopausal woman presented with recurrent syncope episodes for 6 months. Transthoracic echocardiography (TTE) revealed a 6-cm moving structure within the right atrium (**Fig. 1A**). Contrast-enhanced computed tomography (CT) showed a 37-cm-long tumor extending from the right side of the uterine body through the right internal iliac vein and inferior vena cava (IVC) to the right ventricle (**Figs. 1B** and **1C**). There were no abnormalities in her vital signs, electrocardiogram, or routine blood examinations, including the normal D-dimer level of 0.5 μg/ml.

**Figure 2:**
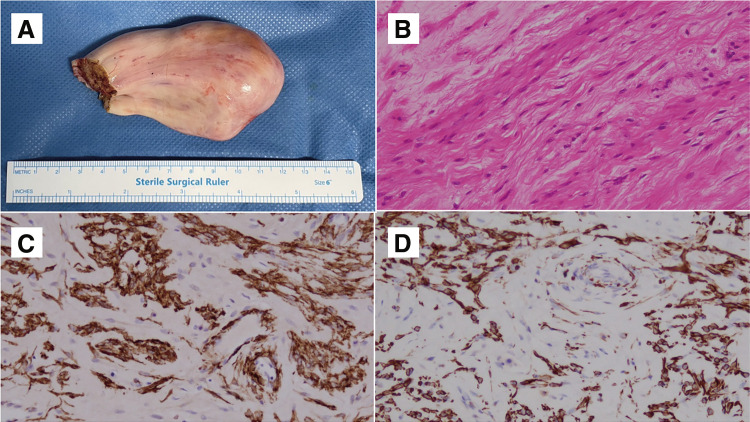
Fig. 2 (**A**) This is a resected tumor, with a smooth surface of approximately 5 cm in diameter and found to be begin macroscopically. (**B**) HE stains. Spindle-shaped cells characteristic of leiomyomatosis are observed. (**C**) Positive for smooth muscle actin. (**D**) Positive for desmin.

**Figure 3:**
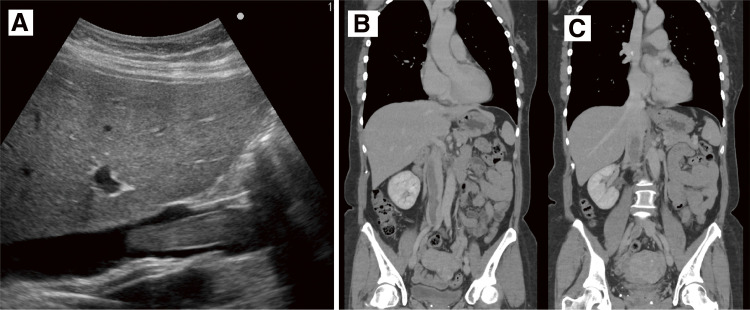
Fig. 3 (**A**) The abdominal ultrasound test revealed a suspicion of tumor growth in size. (**B**, **C**) At the 6-month follow-up CT, the cranial portion of the tumor had grown within the IVC, reaching below the right hepatic vein, raising suspicion of tumor enlargement. IVC: inferior vena cava

**Figure 4:**
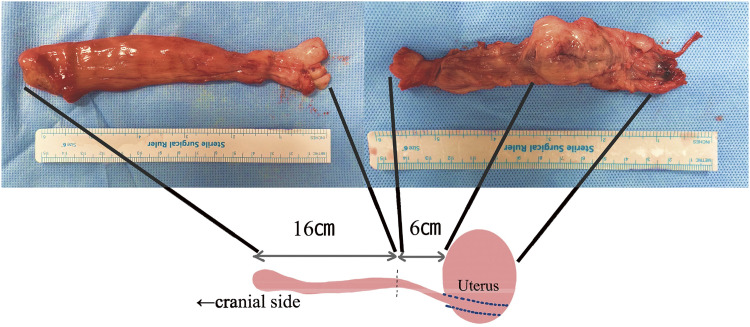
Fig. 4 A resected tumor in the second surgery was shown. The total length of the tumor, including the portion resected during the initial surgery, was 31 cm.

We planned urgent surgery for intracardiac tumor resection. A spherical tumor with a smooth surface of approximately 5 cm in diameter was observed inside the right atrium (**Fig. 2A**). We performed partial resection and left the residual tumor at the hepatic vein level. Histopathological examination confirmed leiomyoma as indicated by the positivity for smooth muscle actin and desmin (**Figs. 2B–2D**). Then we initiated estrogen deprivation therapy for pseudomenopause. Postoperatively, there were no recurrent syncope episodes, and she was discharged on postoperative day 7. The patient was followed up without recurrence or regrowth after the cardiac surgery; however, the 6-month postoperative CT scan revealed a tendency for the uterine leiomyoma to increase in size (**Figs. 3A–3C**). This finding was corroborated by the abdominal ultrasound test, which also suggested tumor growth. Even though the patient had no symptoms after the first surgery, the second-stage surgery to achieve a complete resection of the tumor was conducted jointly by gynecologists and vascular surgeons. Bilateral salpingo-oophorectomy (BSO), total hysterectomy, and intravascular tumor resection were performed (**Fig. 4**). The IVC was incised approximately 4 cm to extract the tumor cranially, but since part of the tumor was adherent to the vessel wall, the right internal iliac vein was also resected en bloc. She had an uneventful postoperative recovery and was discharged on the fifth day after surgery without any anticoagulation therapy. Even though her D-dimer level had mildly increased up to 3.9 μg/ml postoperatively, no significant thrombosis was found that required anticoagulation. We confirmed this finding through a daily ultrasound examination. She has since remained recurrence-free and is being followed up as an outpatient for 6 months. The Institutional Review Board (IRB) number is 20230101, and informed consent was obtained from the patient.

## Discussion

ICLM is a rare condition with limited comprehensive clinical research and its clinical characteristics vary in each case, making it challenging to establish standardized treatment. This patient’s primary complaint was repetitive syncope, and a definitive diagnosis of ICLM was made by TTE and CT. Among ICLM cases, 84% experienced syncope.^3^^)^

Regarding treatment strategies, complete tumor resection is the most effective treatment with lower recurrence rates.^2^^,^^4^^)^ Consequently, discussions often revolve around whether to perform 1-staged complete resection surgery or opt for 2-staged surgery, particularly in cases with firm or extensive adhesions where only partial resection is available. However, although very rare, there are reports^5^^,^^6^^)^ where patients have been followed up with partial resection and postoperative hormone therapy. One case has shown that follow-up aromatase inhibitor administration after partial tumor resection for a menopausal patient resulted in no recurrence for 6 months.^6^^)^

In this case, it was presumed that the tumor invagination to the right ventricle through the tricuspid valve caused a decrease in right cardiac output, leading to syncope. The possibility of the tumor impacting hemodynamics and the potential fatality upon tumor entrapment in the tricuspid valve made our initial decision to resect the intracardiac tumor a less disputable treatment choice.

The surgical resection was performed under extracorporeal circulation, with the right atrium opened to directly observe the tumor. The tumor showed no adhesion to the vessel wall. However, the firm adhesion to the root of the uterine body made its detachment difficult; hence, we partially resected the intracardiac tumor and left the residual part at the hepatic vein level.

Briefly discussing the surgical approach here, the tumor resection had to begin with a right atrial incision, necessitating open-heart surgery performed under the extracorporeal circulation. Intraoperative findings revealed no adhesion between the intracardiac tumor and the vascular walls, including the right atrial wall and the IVC wall; however, this could only be confirmed by opening the heart. Attempting an alternative approach via more caudal veins without the extracorporeal circulation would have been a high risk due to the potential for blind traction on the intracardiac tumor. Although echocardiographic findings showed no apparent adhesion between the tumor and the right atrium, it was considered essential to confirm intraoperatively whether there was any adhesion to the tricuspid valve or the right atrial wall. This surgery was performed under the extracorporeal circulation for these reasons.

Postoperatively, we were concerned about the regrowth of the residual tumor and deliberated whether we should attempt complete resection in a second-stage procedure. In consultation with gynecologists, a decision was made to inhibit leiomyoma growth by hormone therapy (GnRH agonist administration and/or BSO could be a choice because this patient was premenopausal) instead of making a complete resection immediately.

This strategy seemed to be effective for some months, but, unfortunately, during the follow-up CT at 6 months, tumor enlargement was recognized. This finding led us to ultimately proceed with a secondary surgery because hormone therapy alone was not totally effective in controlling tumor growth. As of the 6-month follow-up, the patient remained asymptomatic.

In the intraoperative findings of the 2nd-stage surgery, the uterine leiomyoma extending from the uterus ascended through the right internal iliac vein, continued into the IVC, and reached the right atrium. Consequently, it was necessary to resect the right internal iliac en bloc along with the tumor. One report^7^^)^ indicates that among uterine leiomyomas extending into the heart, approximately 66% reached the heart from the uterus via the iliac veins, about 17% via the ovarian veins, and approximately 7% via both veins. Identifying the roots of venous involvement preoperatively is crucial for determining the surgical strategy.

Finally, we will briefly discuss the decision between a 1-stage (involving gynecologists in the initial surgery) and a 2-stage surgery. Although successful 1-stage complete resections have been reported,^8^^)^ in this case, the strong adhesion between the tumor and the uterus made it highly challenging to achieve complete resection during the initial surgery. Given the urgency to resolve recurrent syncope and the risk of sudden hemodynamic collapse due to tumor entrapment in the tricuspid valve, we prioritized life-saving measures by performing a semi-emergency surgery to remove the intracardiac tumor first. This approach allowed for a safer and more effective resolution of immediate risks while providing time to evaluate and plan the management of the caudal portion of the tumor.

We encountered a case in which hormone therapy following partial resection initially appeared to be effective, but ultimately, complete resection was required. This highlights the importance of complete resection for IVL rather than partial resection followed by hormone therapy and close follow-up.

## Conclusion

We described a case of ICLM. Our palliative surgical resection of the right atrial tumor successfully prevented the repetitive episodes of syncope and reduced the risk of sudden death. The patient was followed only with estrogen deprivation therapy but finally showed the growth of the remaining tumor, which needed to proceed with secondary surgery to achieve complete resection. The complete resection could be the only curative treatment for IVL, even if a partial resection was performed at the time of the initial diagnosis.

## Declarations

### Informed consent

Written informed consent was obtained from the patient to publish this case report and accompanying images.

### Acknowledgments

We would like to thank Tatsuya Shimogawara (Department of Vascular Surgery, Saiseikai Yokohamashi Tobu Hospital, Kanagawa, Japan) and Yasuo Akiba (Department of Obstetrics and Gynecology, Saiseikai Yokohamashi Tobu Hospital, Kanagawa, Japan) for providing advice and Edward F. Barroga for English language editing. No funding was received for this work.

### Submission declaration and verification

This article is original and has not been published elsewhere.

### Disclosure statement

All authors have no conflict of interest.

### Author contributions

Study conception: KK

Data collection: KK

Analysis: none

Investigation: KK

Manuscript preparation: KK

Funding acquisition: none

Critical review and revision: all authors

Final approval of the article: all authors

Accountability for all aspects of the work: all authors.
